# MicroRNA Expression Profiling by Bead Array Technology in Human Tumor Cell Lines Treated with Interferon-Alpha-2a

**DOI:** 10.1007/s12575-009-9012-1

**Published:** 2009-07-23

**Authors:** Fredy Siegrist, Thomas Singer, Ulrich Certa

**Affiliations:** 1 Pharmaceutical Research, Global Preclinical Safety (PRN), F. Hoffmann-La Roche Ltd., 4070, Basel, Switzerland

**Keywords:** MicroRNAs, Oligonucleotide Array Sequence Analysis, Interferons, Melanoma, Hepatoma, Reverse Transcriptase Polymerase Chain Reaction, Suppressor of Cytokine Signaling Proteins

## Abstract

MicroRNAs are positive and negative regulators of eukaryotic gene expression that modulate transcript abundance by specific binding to sequence motifs located prevalently in the 3' untranslated regions of target messenger RNAs (mRNA). Interferon-alpha-2a (IFNα) induces a large set of protein coding genes mediating antiproliferative and antiviral responses. Here we use a global microarray-based microRNA detection platform to identify genes that are induced by IFNα in hepatoma- or melanoma-derived human tumor cell lines. Despite the enormous differences in expression levels between these models, we were able to identify microRNAs that are upregulated by IFNα in both lines suggesting the possibility that interferon-regulated microRNAs are involved in the transcriptional repression of mRNA relevant to cytokine responses.

## 1. Introduction

The gene expression patterns of tumor-derived cell lines differ greatly, as do their responses to antiproliferative effects of interferons (IFNs). The cause of this variation has been under investigation for more than 40 years, but only basic regulatory mechanisms of interferon signaling are understood today. Small regulatory genome encoded RNAs, such as microRNAs, have recently attracted attention in genomic research. New methods to analyze the levels of these regulatory elements are now commercially available, but the power of these techniques is still discussed extensively. Our study was designed to compare two methods for microRNA detection with respect to usefulness in defined cell culture assays. The experimental design assesses variation between the two cell lines and the treatment effects of IFNα.

A hallmark of the therapeutic activity of type I interferons is the induction of antiproliferative activity mediated by the upregulation of several hundred response genes with pleiotropic functions [1]. These genes can be divided into two major classes based on the kinetic properties of induction [2]. Primary response genes (PRGs) are upregulated within 24 h after the cytokine signal and the secondary response genes (SRGs) are induced following day 1 when the activity of the PRGs decays. In contrast to SRGs, all PRGs studied to date contain bona fide interferon response elements in the promoter region, which are required for binding of the interferon-stimulated gene factor 3 (ISGF3) complex and for janus kinase/signal transducer of transcription (JAK/STAT)-pathway-mediated signaling.

Expression of PRGs is turned off by proteins termed suppressors of cytokine signaling (SOCS) [3]. As the nomenclature indicates, this class of polypeptides has the capacity to interfere and silence other cytokine-induced signaling cascades (for review *see *[4]). SOCS1 for instance is part of the early inducible PRG cluster and down modulation occurs together with the other genes before onset of SRG expression. It is believed that feedback inhibition of JAK/STAT signaling by SOCS1 represses transcriptome modulation of IFNα signaling [5]. Regulation of SOCS protein translation by interferon-regulated microRNAs (IRmiRs) would enhance the potential of cytokine fine regulation. It has been reported that miR-19 antagonists lead to higher SOCS1 levels and miR-19 mimics can repress SOCS1 reporter constructs, thus obviously supporting the bioinformatic predictions that SOCS1 is a direct target of miR-19 [6]. Inhibition of SOCS activity could for instance prolong the duration of cytokine activity, which has obvious clinical implications.

Following the discovery of microRNAs in virtually all higher eukaryotic organisms significant research efforts were initiated to address the function of these catalytic oligonucleotides which are the natural counterparts of synthetic small inhibitory RNAs (siRNAs) used for experimental gene silencing (for review *see *[7]). MicroRNAs are positive and negative modulators of the expression of entire gene clusters that contain complementary microRNA recognition sequence motifs in the 3'-UTR. Today, prediction of microRNA target genes by homology-based algorithms is still ambiguous [8]. The activity of one or several microRNAs could explain suppression of the entire PRG cluster provided that microRNA abundance is regulated by IFNα. Alternatively, microRNA-mediated degradation of transcripts encoding negative regulatory proteins would also abolish PRG expression and restore IFNα responsiveness.

Some recent reports showed that interferon beta (IFNβ) stimulation can boost microRNA levels in cell culture together with inhibition of viral replication [9]. At this point it is an open question whether this induction is IFNβ specific or a shared feature of all type I interferons. To investigate whether microRNA are also involved in regulation of IFNα response, we used two human-tumor-derived cell lines: the melanoma line ME-15 [10] and the hepatoma line HuH7 [11]. We have chosen these cell lines as models, because we have a good understanding of the IFNα responses at the mRNA and the protein levels in these cell lines. Further we chose to use a melanoma cell line because IFN is also used for treatment of this cancer type. HuH7 is commonly used as a model for testing antiviral effects of IFN in the HCV replicon system. In both models efficient responses to IFNα have been shown at the functional and transcriptional level. IFNα response genes carry response elements in their promoter region and these motifs are responsible for gene expression with similar efficiency in many cell types. Therefore we expected to find a similar regulated set of genes in both lines given that IRmiR genes are regulated by the same mechanism, whereas some constitutively expressed microRNA genes were expected to be cell type specific for functional reasons. We have chosen a DNA-microarray-based technology (Illumina) for the multi-parallel expression analysis of all known human microRNAs (http://microrna.sanger.ac.uk/; Release 10.0: August 2007). This method allowed us to process total RNA as template, allowing the possibility of mRNA gene expression profiling in further experiments. Briefly, annealing of microRNA specific primers combined with enzymatic polyadenylation allows multi-parallel polymerase chain reaction (PCR)-mediated amplification of individual microRNAs. The output of this step is a DNA amplicon library that reflects to a large extent the original stoichiometry of mature microRNAs in a cell or tissue [12]. PCR amplification is performed with fluorescently labeled primers, which allows quantitative signal detection by conventional confocal laser scanning.

## 2. Materials and Methods

### 2.1. Cell Culture, Interferon Treatment, and RNA Precipitation

Melanoma cells (ME-15) were cultured in RPMI 1640 with L-Glutamine supplemented with non-essential amino acids and sodium pyruvate (1 mM) and hepatoma (HuH7) cells were cultured in DMEM + GlutaMAX. Both media contained 10% FBS. All cell culture reagents were purchased from Invitrogen (GIBCO^®^). Roferon (Interferon alpha2a, ROCHE) was diluted in fresh medium to a final concentration of 1,000 U/mL and control cultures were grown without cytokine. Cells were cultured at 37°C in a humidified atmosphere containing 5% CO_2_. Total RNA preparation was carried out using TRIZOL (Invitrogen) total RNA extraction using 1/2 volume of 1-bromo-3-chloro-propane (molecular biology grade, SIGMA) as chloroform substitute. For efficient recovery of small RNAs, DNA LoBind tubes (Eppendorf) were used and all centrifugation steps were performed at maximum speed and 4°C in an Eppendorf 5417R centrifuge. Total RNA was precipitated with 2 vol of 2-propanol (Fluka) at -20°C for at least 16 h. The RNA pellet was washed with 75% ethanol (Merck), dried, and dissolved in DEPC-treated water (Ambion). The RNA was quantified with Quant-iT™ RiboGreen^®^ RNA Assay (Invitrogen) as suggested by Illumina.

### 2.2. Illumina Bead Array MicroRNA Detection

Starting with 500 ng/sample of total RNA, mature microRNAs were amplified with the Illumina human v1 MicroRNA expression profiling kit containing primers for 743 human microRNAs. The resulting amplicons were hybridized to a 96 sample universal probe capture array and fluorescent signals were detected by confocal laser scanning. All steps were performed according to Illumina's instructions manual.

### 2.3. Data Processing and Statistical Analysis

**Figure 1:**
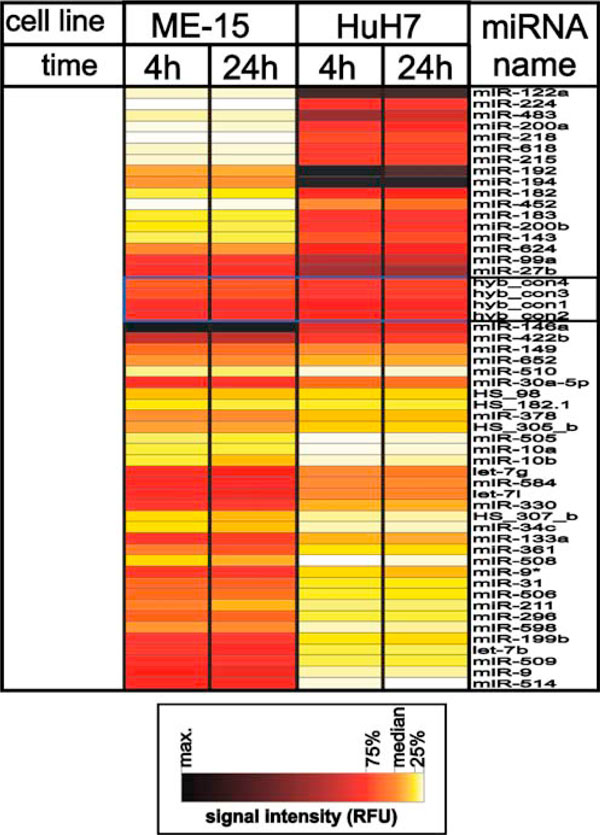
**Differential microRNA expression in human melanoma (ME-15) and hepatoma (HuH7) cells**. microRNA expression levels were compared in two cell lines at two different time points and corrected for the treatment effect. The 50 most significant (*p* value below 10^-12^) microRNA expression values from untreated samples are shown in a heat diagram including hybridization controls as reference for technical variance. *White* indicates noise levels, *yellow* indicates the first quartile, *orange* the median, *red* the third quartile, and *black* maximum expression levels. The intensity data, significance values and the IFNα-dependent expression levels are summarized in Table 1.

**Figure 3:**
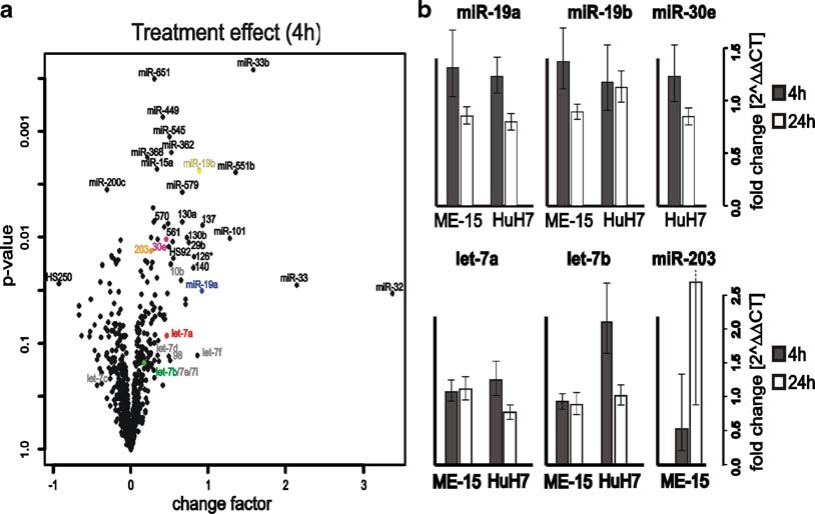
**IFNα-dependent modulation of microRNA expression**. **a** Volcano plot display of IFNα induced microRNA upregulation 4 h after induction. The change factor values (2^log factor change -1) are plotted on the *X*-axis against the *p* value in logarithmic scale on the *Y*-axis. Top-rated microRNAs are annotated together with the let-7 family members. **b** Confirmation of IFNα effect for selected microarray data by qPCR. The CT-values are the average of three technical and three biological replicates and changes were calculated with 2^ΔMNE (mean normalized expression values). *Error bars* show 2^ΔMNE ± Δ*x* (average standard error of treated and untreated MNE) from biological triplicates. miR-30e failed to amplify in ME-15 and miR-203 was below the detection limit in HuH7. Expression values were normalized against endogenous snoRNA RNU48 levels.

The data was processed with Beadstudio software (version 3.1.3, gene expression module 3.3.8) including the calculation of detection *p* values based on negative control bead signals. Log-transformation, loess normalization [13] and statistical analysis were performed with R (2.8.1) [14] using the package lumi (1.8.3) [15] and software contained therein, in particular limma (2.16.4) [16]. Statistical models were chosen as follows: a linear model (limma *t* statistics) with two separate coefficients for HuH7 and ME-15 cells was used for the selection of differently expressed genes shown in Figure 1 and Table 1. Statistics represented in the tables were calculated by testing the two indicated conditions as independent factors. In Table 1, *p* values were adjusted by the false discovery rate method [17]. Treatment effects shown in Table 2b and Figure 3a were modeled with two coefficients (cell line, treatment) for time point 4 h, *p* values arise from *t* statistics. Normalized relative fluorescence levels were calculated by 2^mean (of log2 transformed, loess normalized values). Change factors (CHF) were calculated as fold change on the linear scale minus 1 as previously described [2]. Raw data, non-normalized, and normalized microRNA expression data have been submitted to the Gene Expression Omnibus with accession number GSE16421.

**Table 1:** Cell line differences in microRNA expression

	Control						Interferon-alpha treated					
		
	4 h			24 h			4 h			24 h		
				
	ME-15	HuH7	CHF	ME-15	HuH7	CHF	ME-15	HuH7	CHF	ME-15	HuH7	CHF
microRNAs rated higher in HuH7												
hsa-miR-122a	685	30,912	44.14***	704	32,058	44.51***	717	30,459	41.46***	876	29,044	32.17***
hsa-miR-224	359	10,085	27.07***	345	7,358	20.31***	358	8,183	21.87***	333	7,483	21.44***
hsa-miR-483	915	23,671	24.87***	811	18,073	21.29***	849	17,784	19.95***	802	17,554	20.88***
hsa-miR-200a	517	8,392	15.22***	667	13,543	19.29***	722	11,317	14.68***	500	12,649	24.30***
hsa-miR-218	405	5,654	12.96***	417	6,527	14.64***	419	5,764	12.76***	431	6,155	13.29***
hsa-miR-618	700	7,549	9.78***	649	8,128	11.52***	651	8,042	11.35***	616	7,416	11.03***
hsa-miR-215	638	6,826	9.71***	829	6,013	6.25***	663	6,641	9.02***	723	6,501	7.99***
hsa-miR-192	3,956	34,724	7.78***	3,401	34,181	9.05***	4,021	29,382	6.31***	3,343	33,293	8.96***
hsa-miR-194	4,410	32,950	6.47***	4,142	32,991	6.96***	4,700	33,182	6.06***	4,410	32,422	6.35***
hsa-miR-182	1,575	11,480	6.29 ***	2,541	13,088	4.15***	2,227	12,512	4.62***	3,067	13,907	3.53***
hsa-miR-452	434	2,284	4.27***	707	3,326	3.70***	530	3,378	5.37***	636	3,028	3.76***
hsa-miR-183	1,763	8,748	3.96***	2,123	8,945	3.21***	2,000	8,984	3.49***	2,129	8,258	2.88***
hsa-miR-200b	1,934	7,551	2.90***	1,692	9,633	4.69***	1,705	9,393	4.51***	1,911	10,855	4.68***
hsa-miR-143	1,355	5,087	2.75***	1,775	7,167	3.04***	1,698	5,688	2.35***	1,765	7,519	3.26***
hsa-miR-624	4,852	13,418	1.77**	4,188	11,593	1.77***	4,479	11,718	1.62**	3,872	10,539	1.72**
hsa-miR-99a	8,653	20,232	1.34**	8,887	20,645	1.32***	9,064	18,019	0.99**	8,870	19,506	1.20**
hsa-miR-27b	10,067	22,544	1.24**	11,924	23,914	1.01***	11,564	23,044	0.99***	12,182	24,515	1.01**
microRNAs rated higher in ME-15												
hsa-miR-146a	36,976	15,721	-1.35**	33,410	16,103	-1.07***	35,166	15,137	-1.32***	35,688	19,962	-0.79***
hsa-miR-422b	19,264	7,759	-1.48**	19,962	7,832	-1.55***	20,641	7,462	-1.77***	17,453	7,152	-1.44***
hsa-miR-149	5,868	2,246	-1.61**	6,195	2,030	-2.05***	6,061	1,743	-2.48***	5,477	2,418	-1.26**
hsa-miR-510	1,051	368	-1.86***	1,489	396	-2.76***	1,435	366	-2.92***	1,053	384	-1.74***
hsa-miR-30a-5p	10,664	3,717	-1.87**	10,223	3,700	-1.76***	10,686	3,477	-2.07***	10,466	4,538	-1.31***
HS_98	3,274	1,065	-2.08**	3,225	859	-2.76***	3,445	965	-2.57***	2,850	750	-2.80***
hsa-miR-340	4,583	1,443	-2.18**	4,218	1,220	-2.46***	4,664	1,061	-3.40***	3,078	933	-2.30***
HS_182.1	1,965	615	-2.2**	1,687	606	-1.79**	1,903	562	-2.39***	1,844	534	-2.45***
hsa-miR-378	4,619	1,371	-2.37**	4,975	1,364	-2.65***	4,994	1,213	-3.12***	4,188	1,237	-2.38***
HS_305_b	4,105	1,207	-2.40*	3,853	833	-3.63***	4,278	977	-3.38***	3,688	1,076	-2.43***
hsa-miR-505	1,312	343	-2.83***	2,070	342	-5.05***	2,050	360	-4.70***	2,030	362	-4.60***
hsa-miR-10a	1,570	350	-3.48**	1,592	350	-3.55***	1,828	360	-4.07***	1,880	352	-4.34***
hsa-miR-10b	1,695	378	-3.49**	2,929	371	-6.89***	3,564	411	-7.67***	4,297	382	-10.26***
hsa-let-7g	10,720	2,302	-3.66**	16,046	3,077	-4.21***	13,792	2,854	-3.83***	15,435	2,568	-5.01***
hsa-miR-584	11,643	2,261	-4.15***	12,717	1,873	-5.79***	12,394	1,912	-5.48***	12,616	1,674	-6.54***
hsa-let-7i	11,701	2,255	-4.19**	16,410	2,707	-5.06***	15,319	2,251	-5.80***	15,718	2,563	-5.13***
hsa-miR-330	9,029	1,653	-4.46***	9,684	1,670	-4.80***	9,432	1,397	-5.75***	7,773	1,380	-4.63***
HS_307_b	2,453	444	-4.52***	4,188	443	-8.45***	3,669	407	-8.01***	3,318	448	-6.41***
hsa-miR-34c	2,292	412	-4.56**	3,303	426	-6.75***	3,524	381	-8.25***	2,403	404	-4.95**
hsa-miR-133a	9,270	1,594	-4.82***	7,942	1,662	-3.78***	8,488	1,435	-4.92***	6,495	1,783	-2.64***
hsa-miR-361	5,234	828	-5.32***	7,641	755	-9.13***	7,356	875	-7.41***	7,904	706	-10.19***
hsa-miR-508	2,552	325	-6.86**	4,280	376	-10.39***	3,965	357	-10.09***	4,013	336	-10.96***
hsa-miR-9*	8,372	1,039	-7.06***	10,114	940	-9.76***	10,027	1,263	-6.94***	9,948	843	-10.81***
hsa-miR-31	5,967	661	-8.03***	5,742	720	-6.97***	6,619	657	-9.07***	5,978	728	-7.21***
hsa-miR-506	5,855	625	-8.37***	6,125	713	-7.59***	5,916	665	-7.90***	5,602	675	-7.30***
hsa-miR-211	5,147	476	-9.81***	3,218	490	-5.57***	3,833	471	-7.14**	5,575	464	-11.01***
hsa-miR-296	6,194	570	-9.86***	5,913	504	-10.72***	6,441	557	-10.57***	4,369	532	-7.21***
hsa-miR-598	4,445	401	-10.09***	4,475	386	-10.59***	4,934	424	-10.63***	4,127	367	-10.24***
hsa-miR-199b	9,081	775	-10.71***	9,478	916	-9.35***	9,629	865	-10.13***	8,059	911	-7.85***
hsa-let-7b	8,236	539	-14.28***	12,563	475	-25.44***	12,150	500	-23.30***	12,309	452	-26.24***
hsa-miR-509	10,413	536	-18.44***	10,346	512	-19.22***	9,966	548	-17.20***	9,311	529	-16.62***
hsa-miR-9	12,587	422	-28.85***	15,870	447	-34.50***	15,502	418	-36.09***	16,402	429	-37.26***
hsa-miR-514	12,065	364	-32.16***	13,112	358	-35.64***	12,201	315	-37.74***	12,355	355	-33.83***
Hybdridization controls												
array_hyb_con4	6,404	7,110	0.11	7,724	6,684	-0.16	7,031	6,739	-0.04	6,964	6,746	-0.03
array_hyb_con3	6,923	7,681	0.11	7,833	7,368	-0.06	7,422	7,054	-0.05	6,906	7,365	0.07
array_hyb_con1	10,779	11,868	0.10	11,812	11,728	-0.0.01	11,436	11,382	0.00	11,059	11,522	0.04
array_hyb_con2	10,258	11,145	0.09	11,382	10,829	-0.05	11,075	10,650	-0.04	9,997	10,283	0.03

List of calculated relative fluorescent levels (without background correction) and calculated differences (CHF) between hepatoma (HuH7) and melanoma (ME-15) cells. A linear model with ME-15 and HuH7 as separate factors was used to estimate cell line differences in microRNA expression. The genes with the highest significance (and log factor change >0.5) are listed in the upper section and the bottom shows data for the hybridization controls as reference. Change factors show the cell line differences of the retransformed mean to the linear scale. *p* values (limma *t* test) were adjusted by false discovery rate correction. Significance codes are defined by the intervals: '***' < 0.001 ≤ '**' < 0.01 ≤ '*' < 0.05

**Table 2:** Modulation of microRNA expression by IFNα—4 and 24 h after stimulation

	Control						Interferon-alpha treated					
		
	4 h			24 h			4 h			24 h		
				
a	ME-15	HuH7	CHF	ME-15	HuH7	CHF	ME-15	HuH7	CHF	ME-15	HuH7	CHF
Validated microRNAs												
hsa-miR-19a	13,617	6,726	-1.02	18,318	17,178	-0.07	19,226	17,409	-0.10	14,425	14,079	-0.02
hsa-miR-19b	13,365	9,406	-0.42	25,463	22,438	-0.13	21,532	20,625	-0.04	18,039	17,440	-0.03
hsa-miR-30e-5p	9,497	6,838	-0.39	13,045	11,321	-0.15	12,643	10,887	-0.16	13,230	11,809	-0.12
hsa-let-7a	15,244	4,335	-2.52.	28,304	6,181	-3.58 ***	22,226	6,386	-2.48***	30,449	5,612	-4.43***
hsa-let-7b	8,236	539	-14.28***	12,563	475	-25.44***	12,150	500	-23.30 ***	12,309	452	-26.24***
hsa-miR-203	907	325	-1.79**	1,155	348	2.32***	1,302	359	-2.63***	1,186	5,757	-1.07**
hsa-miR-130b	5,700	8,316	-0.46	13,023	13,880	0.07	9,994	14,055	0.41	12,053	12,432	0.03
hsa-miR-455	2,677	2,604	-0.03	3,944	5,285	0.34*	3,437	5,927	0.72**	3,262	5,342	0.64*
b	ME-15						HuH7					
	4 h			24 h			4 h			24 h		
	-IFNa	+IFNa	CHF	-IFNa	+IFNa	CHF	-IFNa	+IFNa	CHF	-IFNa	+IFNa	CHF
Interferon-regulated microRNAs												
hsa-miR-33b	1,226	3,484	1.84**	2,888	2,318	-0.25	476	1,113	1.34**	1,306	1,851	0.42***
hsa-miR-33	2,631	7,352	1.79	9,774	5,764	-0.70*	1,887	6,645	2.52.	8,893	2,726	-2.26
hsa-miR-126*	2,221	5,409	1.44*	5,578	6,111	0.10	4,749	6,410	0.35.	5,687	5,803	0.02***
hsa-miR-10b	1,695	3,564	1.10*	2,929	4,297	0.47**	378	411	0.09	371	382	0.03
hsa-miR-551b	2,085	4,169	1.00*	4,423	3,419	-0.29	1,804	4,979	1.76*	5,221	4,865	-0.07
hsa-miR-137	1,037	1,966	0.90	2,523	2,872	0.14	1,613	3,155	0.96**	3,429	3,598	0.05
hsa-miR-138	2,158	4,074	0.89*	4,709	3,701	-0.27	725	828	0.14	903	859	-0.05
hsa-miR-130b	5,700	9,994	0.75	13,023	12,053	-0.08	8,316	14,055	0.69**	13,880	12,432	-0.12
hsa-miR-101	6,701	11,387	0.70	13,088	11,688	-0.12	3,569	10,871	2.05**	10,445	10,796	0.03
hsa-miR-140	7,339	12,236	0.67	15,512	15,931	0.03	3,058	5,976	0.95*	6,135	7,221	0.18
HS_92	829	1,356	0.64.	1,246	1,179	-0.06	407	587	0.44	492	478	-0.03
hsa-miR-362	1,382	2,234	0.62*	2,144	2,276	0.06	1,907	2,737	0.43*	2,456	2,519	0.03
hsa-miR-19b	13,365	21,532	0.61**	25,463	18,039	-0.41	9,406	20,625	1.19*	22,438	17,440	-0.29
hsa-miR-130a	13,031	20,878	0.60.	27,571	23,048	-0.20	17,884	31,012	0.73*	33,801	28,875	-0.17
hsa-miR-579	552	816	0.48.	992	1,053	0.06	729	1,362	0.87*	1,250	1,308	0.05
hsa-miR-29b	23,138	34,148	0.48.	32,691	29,710	-0.10	8,737	17,989	1.06*	20,599	17,405	-0.18.
hsa-miR-19a	13,617	19,226	0.41*	18,318	14,425	-0.27	6,726	17,409	1.59.	17,178	14,079	-0.22.
hsa-miR-338	1,298	1,813	0.40.	1,870	1,603	-0.17	2,363	4,603	0.95.	5,109	4,088	-0.25
hsa-miR-590	1,403	1,949	0.39.	1,545	1,367	-0.13	669	1,068	0.60.	884	847	-0.04
hsa-miR-545	1,455	1,973	0.36.	2,149	1,720	-0.25*	829	1,371	0.65**	1,573	1,393	-0.13
hsa-miR-30e-5p	9,497	12,643	0.33	13,045	132,030	0.01	6,838	10,887	0.59*	11,321	11,809	0.04
hsa-miR-570	1,989	2,576	0.30.	2,397	2,610	0.09	2,213	3,739	0.69*	4,312	3,708	-0.16
hsa-miR-301	13,621	17,531	0.29*	16,321	15,142	-0.08	5,646	10,460	0.85*	10,925	10,295	-0.06.
hsa-miR-561	517	621	0.20	568	464	-0.22**	683	1,157	0.69**	1,149	941	-0.22
HS_250	4,284	1,813	-1.36.	740	983	0.33	3,688	2,337	-0.58	1,195	1,303	0.09
c	ME-15						HuH7					
	4 h			24 h			4 h			24 h		
	-IFNa	+IFNa	CHF	-IFNa	+IFNa	CHF	-IFNa	+IFNa	CHF	-IFNa	+IFNa	CHF
Validated microRNAs												
hsa-miR-19a	13,617	19,226	0.41*	18,318	14,425	-0.27	6,726	17,409	1.59.	17,178	14,079	-0.22
hsa-miR-19b	13,365	21,532	0.61**	25,463	18,039	-0.41	9,406	20,625	1.19*	22,438	17,440	-0.29.
hsa-miR-30e-5p	9,497	12,643	0.33	13,045	13,230	0.01	6,838	10,887	0.59*	11,321	11,809	0.04
hsa-let-7a	15,244	22,226	0.46	28,304	30,449	0.08	4,335	6,386	0.47.	6,181	5,612	-0.10
hsa-let-7b	8,236	12,150	0.48*	12,563	2,309	-0.02	539	500	-0.08	475	452	-0.05
hsa-miR-203	907	1,302	0.44*	1,155	1,186	0.03	325	359	0.10	348	575	0.665**

Microarray data for RT-PCR validated microRNAs, *see* description of Table 1. Average relative fluorescent levels and their change factors (CHFs) are shown for hepatoma (HuH7) and melanoma (ME-15) cells at two time points. Genes were selected by significance calculated in a linear model with interaction assuming that the change may not occur in both cell lines (e.g. miR-10b has no detectable levels in HuH7). *p* value significance codes are defined by the intervals: '***' < 0.001 ≤ '**' < 0.01 ≤ '*' < 0.05 ≤ '.' < 0.1. Section c shows the microarray expression levels of microRNAs assayed by RT-PCR (treatment analysis)

### 2.4. Quantitative PCR and Data Processing

microRNA levels were measured using TaqMan^®^ microRNA assays (Applied Biosystems) using the TaqMan^®^ MicroRNA reverse transcription (RT) kit with TaqMan^®^ 2× universal PCR master mix (No AmpErase^®^ UNG) as recommended by the supplier. Ten nanograms of total RNA was used as input for amplification using the samples used for microarray analysis. Reversed transcriptase products were diluted 1:15 and measured on an ABI 7900HT fast real-time PCR system. Technical replicates were run on three different plates (one with 40 cycles and two with 50 cycles) and threshold for cycling time (CT) calculation was set for all probes to 0.2. For estimation of endogenous small RNA content, the nucleolar RNA RNU48 was used as control and reference.

Standard error (Δ*x*) was calculated by the average standard error of treated and untreated MNE for biological replicates.

## 3. Results and Discussion

The following technical aspects have to be considered for result interpretation. The dataset of the microRNA bead array assay is not directly comparable to gene expression arrays where in vitro translated transcripts are directly hybridized to the probes. Moreover Illumina's bead array technology tends to have higher background fluorescence levels and lower change factor values than Genechips from Affymetrix. Background (average of negative control signals) and noise (standard deviation of negative probes from each sample) were 528 ± 60 and 229 ± 67, respectively. The density of all samples shows a bimodal distribution peaking around the background fluorescent levels and the robust levels (approximately 12,000). The curve is skewed to the right and peak density height is found in the ratio 4:1 considering all probes (data not shown). The distribution of probes detected in all samples (detection *p* value threshold at 0.01) has a plateau ranging from about 2,000 close to the detectors maximum capacity of 2^16 relative fluorescent units [12]. As expected, the correlation of data coming from biological replicates *r*^2^ = 0.952 ± 0.028 (not normalized) and *r*^2^ = 0.956 ± 0.022 (after loess normalization and log-transformation) was lower than for technical replicates *r*^2^ > 0.97 [12]. We preferred loess normalization to quantile normalization because the later was too aggressive for the given small probe numbers.

As a first step we wished to address the robustness of the microRNA array in probe detection by selecting microRNA genes that are detected under all experimental conditions with high statistical significance in all biological triplicates (detection *p* value < 0.01). In each set of triplicate samples (control, 4 h or 24 h, IFNα stimulation) we detect approximately 270 genes that fulfill the above criteria. This corresponds to roughly a third of microRNAs available for detection in the assay system. Furthermore, this result suggests indirectly that IFNα treatment does not induce global changes in microRNA gene expression, but it modulates rather the expression of individual genes.

**Figure 2:**
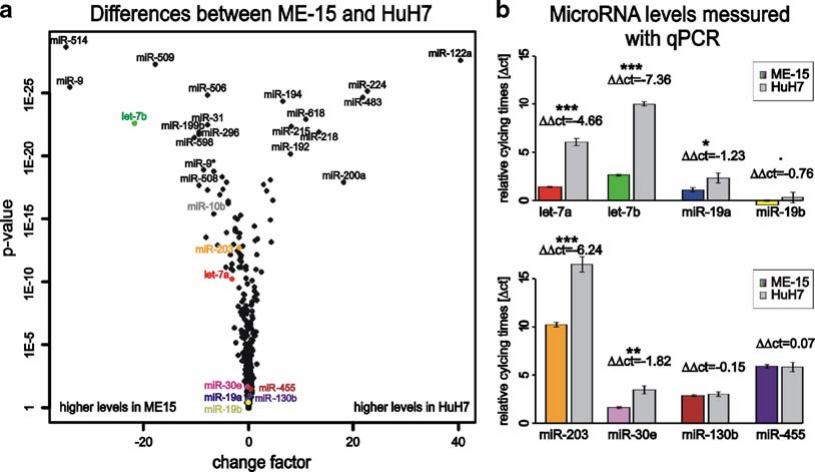
**Cell line specific microRNA expression Volcano plot (**a**) display demonstrates the multi-variant biological diversity of microRNA expression in ME-15 or HuH7 cells**. The estimated fold-change value (change factor) is plotted on the *X*-axis against the *p* value (limma *t* statistics) in logarithmic scale on the *Y*-axis. A linear model, using the expression values of untreated ME-15 cells at 4 h as base together with three parameters to estimate differences in time, treatment, or cell line. The top ranked and qPCR measured microRNAs are annotated. **b** Quantitative PCR validation of microarray data using eight selected microRNAs. Input total RNA came from independent cell cultures. Data are shown as relative cycling times (ΔCT) calculated with endogenous control RNU18 for ME-15 (*color-filled bars*) and HuH7 (*gray*). *Error bars* represent ΔCT ± Δ*x* (standard deviation of biological replicates). ΔΔCT are noted above the bars together with the significance codes for *t* statistics defined by the intervals '***' < 0.001 ≤ '**' < 0.01 ≤ '*' < 0.05 ≤ '.' < 0.1.

Today it is well established that microRNA expression patterns are cell and tissue type specific, which is consistent with a role in cell differentiation and biological function [8]. Thus we expected to detect genes with preferential expression in either hepatoma or melanoma cells as these cell lines are derived from different tumors. Indeed, when all experimental conditions and data points are included in the data analysis about 150 microRNAs genes show preferential expression in either HuH7 or ME-15 cells (Figure 1). Table 1 shows the expression data for the most significant genes including change factors and significance score as reference. Among these differentially expressed genes there are three members of the let-7 family, which has properties of tumor suppressor genes (for review *see *[18]). Therefore it is not surprising that the members of this well-known microRNA gene family are deregulated in the analyzed cancer cells too. Furthermore, the different developmental stage of our cancer cell lines is expected to have left a genomic fingerprint where some microRNA genes are expressed in one but not the other cell line [19]. Consistent with this, expression of some microRNAs is strictly cell type specific and barely detectable in the other cell type (Figure 2a), for example the liver-specific miR-122a and miR-192 [20].

Bead-array-based microRNA detection technology, including the bio-statistic analysis, is currently not well established or widely used and we have applied a commercial PCR-based assay to confirm the array data for some microRNAs that cover different expression levels and change factors. In contrast to mRNA profiling, where RT-PCR-based assays are considered as gold standard for data validation, new generation deep sequencing is considered as the method of choice for microRNA quantification but is not available in our research institute. For the microRNAs let-7 a/b, miR-19 a/b, and miR-203, the PCR-based quantification method (Figure 2b) confirmed the direction of change found with microarray technology (Table 2a). Expression of miR-130b and miR-455 was at similar levels in both assays. The correlation calculated for the eight tested microRNAs was acceptable: multiple *r*^2^ from *f* test of mean relative cycling times (ΔCT) to mean log2 microarray expression values was 0.9279. Differences of absolute levels between the microRNA targets probably results from different hybridization properties of the microarray probes and variation in the performance of Taqman primers for the specific microRNA on the other side.

Assuming that any IFNα relevant microRNA will have the same kinetics as the mRNA for PRGs, we looked at the regulation of microRNA genes in our experiment. These IRmiRs should respond to IFNα stimulation preferentially in both cell lines, because this would be a good indication of a general mechanism in the IFNα response. Within the 25 most significantly regulated genes (Table 2b), only one gene (HS_250) is downregulated. A general upregulation of transcripts is consistent with classic IFNα signaling seen for mRNAs. However, the maximal observed change factor with high significance was 1.84 (miR-33b in Table 2b) which is clearly lower than the values seen for protein coding mRNAs [2]. We also included an expression analysis 24 h after IFNα stimulation in order to detect microRNA genes that show either delayed induction or remain activated at comparable levels to the 4 h stimulus. Based on our data set, the majority of the microRNA response genes show no further induction, but rather moderate downregulation 24 h after induction. This finding is not surprising as we expected immediate early impact of IFNα-mediated primary signaling.

We also measured the IFNα response in the same experiment and for the same microRNAs (Table 2c). When we analyze the IFNα effect at the early time point in both cell lines we find all the validated microRNAs to be upregulated (Figure 3a). The magnitude of upregulation and the basal expression levels of the microRNA-19a and 19b are similar in both cell lines (Figure 3b, top). This and the finding that miR-19 regulates SOCS1 [4] may be relevant for the regulation of cytokine signaling. let-7a and let-7b had higher levels in the melanoma-cell-line-derived samples compared HuH7, but the induction by IFNα in ME-15 could not be reproduced by RT-PCR (Figure 3b, bottom). In both assays accurate fold changes are difficult to calculate, if the baseline expression level is close to background noise or the detection limit. An example of a gene at the detection limit is miR-203, which is not detectable without IFNα treatment in HuH7 cells (Table 2c). Upon IFNα stimulation (24 h in HuH7) the microRNA is detectable above background suggesting minimal induction. Consequently a solid change factor cannot be calculated, which is consistent with the high variance obtained by qPCR (ME-15). This result is in fact not surprising, because both technologies rely on logarithmic PCR amplification of microRNA templates. At low expression levels, both technologies show relatively high variation in biological replicates, which should be considered for data interpretation. Interestingly, miR-203 has a putative binding site for ISGF3 in the promoter region, which would enable IFNα-dependent upregulation. miR-30 has been reported to be IFNβ inducible, although the subclass measured was not specified by the authors [9]. We decided to analyze the most promising candidate (miR-30e-5p) present in our microarray dataset (Figure 3a in gray). Detection of miR-30e failed in ME-15 cells due to technical problems, but induction in HuH7 was similar to miR-19a/b.

Some technology-related questions remain open. The microRNA assay measures essentially the number of amplicons generated by RT-PCR for each transcript. Thus the signal is an indirect measurement of transcript abundance as compared to classical mRNA microarray platforms, where the target mRNA is directly labeled during linear amplification by in vitro transcription. As a consequence, change factor calculations for amplicon-based assays are ambiguous.

In summary, Illumina's bead array technology is well suited for multi-parallel profiling of microRNAs expressed in different cell types or tissues. We were also able to detect IFNα-inducible microRNA genes although the changes observed were moderate and biological significance remains to be proven. Like most microarray-based detection technologies the technical variability among identical samples is low compared to biological variations of individual cell cultures. At this point it is important to note that variation among biological samples occurs and is independent of the parameters that are measured. Consistent with IFNα-dependent induction of mRNAs we find that virtually all modulated microRNA genes are upregulated. However, the IFNα-induced changes detected in our study are relatively small compared to the changes induced by IFNβ in HuH7 cells [9]. Finally, it is noteworthy that IRmiRs have similar kinetic properties to their mRNA counterparts. miR-10b for instance is induced early in ME-15 and remains upregulated, while miR-19 abundance ceases after 24 h. In general, the majority of IRmiR genes were reset to basal levels after 24 h and further studies are needed for kinetic classification. Thus, our study adds another level of complexity to the dynamic regulation IFNα signaling and other mechanisms like epigenetic promoter methylation are currently under intense investigation in our laboratories.

## References

[B1] Borden EC Sen GC Uze G Silverman RH Ransohoff RM Foster GR Interferons at age 50: past, current and future impact on biomedicine Nat Rev Drug Discov 2007 6 12 975 990 10.1038/nrd2422 18049472 18049472 PMC7097588

[B2] Certa U Wilhelm-Seiler M Foser S Broger C Neeb M Expression modes of interferon-alpha inducible genes in sensitive and resistant human melanoma cells stimulated with regular and pegylated interferon-alpha Gene 2003 315 79 86 10.1016/S0378-1119(03)00722-4 14557067 14557067

[B3] Zimmerer JM Lesinski GB Kondadasula SV Karpa VI Lehman A Raychaudhury A IFN-alpha-induced signal transduction, gene expression, and antitumor activity of immune effector cells are negatively regulated by suppressor of cytokine signaling proteins J Immunol 2007 178 8 4832 4845 17404264 17404264 10.4049/jimmunol.178.8.4832

[B4] Yoshimura A Naka T Kubo M SOCS proteins, cytokine signalling and immune regulation Nat Rev Immunol 2007 7 6 454 465 10.1038/nri2093 17525754 17525754

[B5] Song MM Shuai K The suppressor of cytokine signaling (SOCS) 1 and SOCS3 but not SOCS2 proteins inhibit interferon-mediated antiviral and antiproliferative activities J Biol Chem 1998 273 52 35056 35062 10.1074/jbc.273.52.35056 9857039 9857039

[B6] Pichiorri F Suh SS Ladetto M Kuehl M Palumbo T Drandi D MicroRNAs regulate critical genes associated with multiple myeloma pathogenesis Proc Natl Acad Sci U S A 2008 105 35 12885 12890 10.1073/pnas.0806202105 18728182 18728182 PMC2529070

[B7] Bartel DP MicroRNAs: genomics, biogenesis, mechanism, and function Cell 2004 116 2 281 297 10.1016/S0092-8674(04)00045-5 14744438 14744438

[B8] Friedman RC Farh KK Burge CB Bartel D Most mammalian mRNAs are conserved targets of microRNAs Genome Res 2009 19 1 92 105 10.1101/gr.082701.108 18955434 18955434 PMC2612969

[B9] Pedersen IM Cheng G Wieland S Volinia S Croce CM Chisari FV Interferon modulation of cellular microRNAs as an antiviral mechanism Nature 2007 449 7164 919 922 10.1038/nature06205 17943132 17943132 PMC2748825

[B10] Lüscher U Filgueira L Juretic A Zuber M Lüscher NJ Heberer M The pattern of cytokine gene expression in freshly excised human metastatic melanoma suggests a state of reversible anergy of tumor-infiltrating lymphocytes Int J Cancer 1994 57 4 612 619 10.1002/ijc.2910570428 8181865 8181865

[B11] Nakabayashi H Taketa K Miyano K Yamane T Sato J Growth of human hepatoma cells lines with differentiated functions in chemically defined medium Cancer Res 1982 42 9 3858 3863 6286115 6286115

[B12] Chen J Lozach J Garcia EW Barnes B Luo S Mikoulitch I Highly sensitive and specific microRNA expression profiling using BeadArray technology Nucleic Acids Res 2008 36 14 e87 10.1093/nar/gkn387 18579563 18579563 PMC2504321

[B13] Bolstad B Irizarray R Astrand M Speed T A comparison of normalization methods for high density oligonucleotide array data based on bias and variance Bioinformatics 2003 19 185 193 10.1093/bioinformatics/19.2.185 12538238 12538238

[B14] R Development Core Team R: A language and environment for statistical computing 2008 Vienna, Austria: R Foundation for Statistical Computing http://www.R-project.org

[B15] Du P Kibbe WA Lin SM Lumi: a pipeline for processing Illumina microarray Bioinformatics 2008 24 13 1547 1548 10.1093/bioinformatics/btn224 18467348 18467348

[B16] Smyth GK Gentleman R, Carey V, Dudoit S, Irizarry R, Huber W Limma: linear models for microarray data Bioinformatics and computational biology solutions using R and bioconductor 2005 Springer, New York 397 420 10.1007/0-387-29362-0_23

[B17] Benjamini Y Hochberg Y Controlling the false discovery rate: a practical and powerful approach to multiple testing J Roy Stat Soc B Stat Meth 1995 57 1 289 300

[B18] Roush S Slack FJ The let-7 family of microRNAs Trends Cell Biol 2008 18 10 505 516 10.1016/j.tcb.2008.07.007 18774294 18774294

[B19] Gaur A Jewell DA Liang Y Ridzon D Moore JH Chen C Characterization of microRNA expression levels and their biological correlates in human cancer cell lines Cancer Res 2007 67 6 2456 2468 10.1158/0008-5472.CAN-06-2698 17363563 17363563

[B20] Baskerville S Bartel DP Microarray profiling of microRNAs reveals frequent coexpression with neighboring miRNAs and host genes RNA 2005 11 3 241 247 10.1261/rna.7240905 15701730 15701730 PMC1370713

